# Arbuscular mycorrhizal fungi promote coexistence and niche divergence of sympatric palm species on a remote oceanic island

**DOI:** 10.1111/nph.14850

**Published:** 2017-10-16

**Authors:** Owen G. Osborne, Rishi De‐Kayne, Martin I. Bidartondo, Ian Hutton, William J. Baker, Colin G. N. Turnbull, Vincent Savolainen

**Affiliations:** ^1^ Department of Life Sciences Imperial College London Ascot SL5 7PY UK; ^2^ Royal Botanic Gardens, Kew Richmond TW9 3DS UK; ^3^ Lord Howe Island Museum Lord Howe Island NSW 2898 Australia; ^4^ University of Johannesburg Auckland Park Johannesburg 2006 South Africa

**Keywords:** coexistence, ecological speciation, edaphic adaptation, mycorrhizae, Palmae, symbiosis, sympatric speciation

## Abstract

- Microbes can have profound effects on their hosts, driving natural selection, promoting speciation and determining species distributions. However, soil‐dwelling microbes are rarely investigated as drivers of evolutionary change in plants.
- We used metabarcoding and experimental manipulation of soil microbiomes to investigate the impact of soil and root microbes in a well‐known case of sympatric speciation, the *Howea* palms of Lord Howe Island (Australia). Whereas *H. forsteriana* can grow on both calcareous and volcanic soils, *H. belmoreana* is restricted to, but more successful on, volcanic soil, indicating a trade‐off in adaptation to the two soil types.
- We suggest a novel explanation for this trade‐off. Arbuscular mycorrhizal fungi (AMF) are significantly depleted in *H. forsteriana* on volcanic soil, relative to both *H. belmoreana* on volcanic soil and *H. forsteriana* on calcareous soil. This is mirrored by the results of survival experiments, where the sterilization of natural soil reduces *Howea* fitness in every soil–species combination except *H. forsteriana* on volcanic soil. Furthermore, AMF‐associated genes exhibit evidence of divergent selection between *Howea* species.
- These results show a mechanism by which divergent adaptation can have knock‐on effects on host–microbe interactions, thereby reducing interspecific competition and promoting the coexistence of plant sister species.

## Abstract 2

See also the Commentary on this article by Geml & Wagner, **217**: 965–967.

## Introduction

Species diversity is governed by the formation, coexistence and extinction of species. Mechanisms of speciation have received widespread attention in recent years (Wu, 2001; Rieseberg *et al*., 2003; Seehausen, 2004; Barluenga *et al*., 2006; Savolainen *et al*., 2006; Jones *et al*., 2012; Riesch *et al*., 2017). However, understanding the mechanisms of coexistence is also key to explaining patterns of diversity. Two species inhabiting identical niches represent an unstable situation, which can be resolved either by the extinction of one of the species, or by niche differentiation to allow their coexistence (McArthur & Levin, 1967; Chesson, 2000). Consequently, when sister species continue to occupy the same geographic area, divergent adaptation is expected, either during sympatric or parapatric speciation, or following secondary contact.

One of the most compelling cases of sympatric speciation is found in the palm genus *Howea* (Savolainen *et al*., 2006). The two sister species, *H. belmoreana* and *H. forsteriana*, are endemic to the minute and isolated Lord Howe Island (LHI) in the Tasman Sea between Australia and New Zealand. Their speciation scenario involves adaptation to the main soil types on LHI, older volcanic rocks and Pleistocene calcareous deposits (calcarenite), which is thought to have triggered flowering time differences (Savolainen *et al*., 2006; Dunning *et al*., 2016). Given that the two soil types are intercalated on the island, and that the two palms are wind pollinated, speciation most likely happened in the face of high gene flow (Savolainen *et al*., 2006; Babik *et al*., 2009; Papadopulos *et al*., 2013; Dunning *et al*., 2016). Currently, reproductive isolation by flowering time differences is the main barrier to gene flow (Savolainen *et al*., 2006; Hipperson *et al*., 2016). However, there is also evidence of post‐zygotic isolation: although very few hybrids are found on the island, these are more often juveniles than adult trees (Savolainen *et al*., 2006; Hipperson *et al*., 2016). Furthermore, although the species show some evidence of niche differentiation, their ranges substantially overlap, leading to an expectation of competition between the species. Specifically, *H. forsteriana* is found on both alkaline calcareous soil as well as the more acidic volcanic soils. By contrast, *H. belmoreana* is restricted to the volcanic soils. These soils are the main two found on LHI, and *Howea* covers most of the island vegetation. If *H. forsteriana* is outcompeting *H. belmoreana* on volcanic soils, it may drive it to extinction. However, *H. belmoreana* is found to be more common on volcanic soil (Savolainen *et al*., 2006) and, in an island‐wide transplant experiment, *H. belmoreana* also showed higher germination and survival rate on volcanic soil relative to *H. forsteriana* (Hipperson *et al*., 2016). Although there was no significant difference between seedling growth of the two species on calcareous soil (Hipperson *et al*., 2016), the absence of adult *H. belmoreana* on calcareous soil indicates that there is strong selection operating at late stages.

Therefore, soil has clearly played a key role in speciation and remains important for species coexistence. A large transcriptomic study found that only three loci were differentially expressed between the roots of *H. forsteriana* on volcanic vs calcareous soils, but they were of unknown function (Dunning *et al*., 2016). Previous studies of the soil itself have focused on its chemical composition, pH, salinity and soil water content (Savolainen *et al*., 2006; Papadopulos *et al*., 2013); although these abiotic factors differ between the soil types, they offer limited explanatory power regarding the selective pressures that operate on the two species when occupying the same soil type (Papadopulos *et al*., 2013). Here, we focus on one aspect of soil biology that has been neglected, that is, the biotic selection pressure elicited by microbes present in the soil and roots of *Howea*.

Microbial communities can have multiple effects on plants, ranging from disease threatening species survival (Smith *et al*., 2006) to mycorrhizal associations being essential for the completion of the plant life cycle (Curtis, 2012). Many such interactions are poorly understood, and rarely studied in the context of speciation (but see Waterman *et al*., 2011; Ren *et al*., 2016). Microbial communities are known to affect several aspects of plant biology potentially relevant to *Howea*. For example, microbes alter flowering time in *Boechera stricta* and *Arabidopsis thaliana* (Wagner *et al*., 2014; Panke‐Buisse *et al*., 2015), affecting the patterns of selection in the former species. Mycorrhizal and bacterial symbionts are known to allow plant growth on nutrient‐poor soil by delivering phosphorus and nitrogen in exchange for carbohydrates produced by the plant (Denison & Kiers, 2011). Indeed, associations with different mycorrhizal fungi have been shown to promote coexistence in orchids (Waterman *et al*., 2011). Pathogens are also likely to influence the distribution of plant species, with pathogens known to be less effective against plants outside their native range (Mitchell & Power, 2003).

In this study, we used a ribosomal DNA‐based metabarcoding approach on the roots and surrounding soil of *Howea*. We compared the fungal and bacterial communities in both species (*H. forsteriana* and *H. belmoreana*) and soil types (volcanic and calcareous). Using survival experiments, we tested whether the soil microbiome affects seedling fitness. These data allow us to test the hypothesis that microbes have an impact on species coexistence and to evaluate their role in the evolution of *Howea*.

## Materials and Methods

### Sample collection

**Figure 1:**
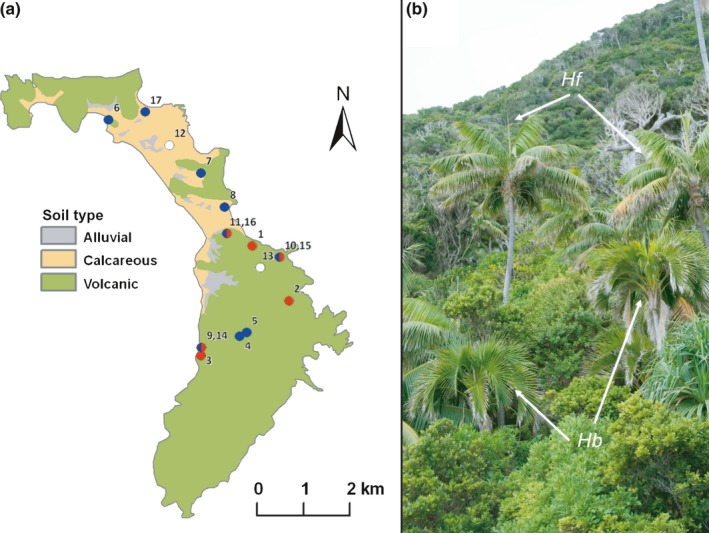
(a) Broad‐scale geological map of Lord Howe Island showing sampling localities. Calcareous soil is shown in yellow, alluvial soil in grey and volcanic soil in green. Monotypic *Howea belmoreana* sites are represented by red circles, monotypic *H. forsteriana* sites by blue circles, sites with both species by red and blue circles, and those with no *Howea* by white circles. Note that the sites that contained both species received different site numbers for the different species although the location was the same. (b) *Howea forsteriana* (*Hf*) and *H. belmoreana* (*Hb*) growing wild on Lord Howe Island.

Soil and root samples for microbial community analysis were collected from 17 sites across LHI (Fig. 1). These comprised paired root and soil samples from six sites with *H. belmoreana* growing on volcanic soil (root : B_root_ and soil : B_soil_), four sites with *H. forsteriana* on calcareous soil (root : F_cal‐root_ and soil : F_cal‐soil_) and five sites with *H. forsteriana* on volcanic soil (root : F_vol‐root_ and soil : F_vol‐soil_; three of these sites also had *H. belmoreana* present, which we also collected for analysis; they are shown in Fig. 1a as red–blue circles; GPS coordinates are listed in Supporting Information Table S1). We also collected soil where no palms were present: (N_vol‐soil_ and N_cal‐soil_; Table S1). At each of these sites (excluding the two with no *Howea*), five adult trees at least 1 m apart were sampled. For each tree, one 5‐cm soil core (*c*. 25 cm^3^) was removed immediately adjacent to the base of the tree, beginning at a depth of 20 cm. Each soil sample was then homogenized by hand and a subsample of 4 g was taken from each, mixed with RNAlater (Qiagen, Valencia, CA, USA) and frozen at −20°C to halt microbial growth. In addition to soil, palm roots were collected at each site. From each of the five trees, a 10‐cm‐long section of root was collected. Root samples were washed in sterile water to remove soil. Approximately 2 cm of each root sample was reserved for microscopy; this was fixed in 70% ethanol : 8% acetic acid (3 : 1, v/v) for 24 h before draining and storing at room temperature. The remainder of each sample was cut into 5‐mm pieces before being frozen in RNAlater for DNA extraction.

### DNA extraction and sequencing

Soil DNA extractions were carried out using the ZR‐96 Soil Microbe DNA Kit (Zymo Research, Orange, CA, USA) following the manufacturer's instructions using *c*. 0.8 g of soil per sample. Root DNA extractions were carried out using the Qiagen DNeasy Plant Mini Kit (Qiagen, Valencia, CA, USA) following the manufacturer's instructions with *c*. 0.5 g of root tissue and including the optional addition of RNase‐A to remove unwanted RNA. The five DNA extracts for each sample type (root or soil) from each site were then pooled, resulting in 32 samples. Samples were sent to MrDNA (Shallowater, TX, USA) for PCR amplification, library construction and sequencing. Both the V4 hyper‐variable region of the 16S ribosomal subunit and the ITS2 region were PCR amplified to characterize the communities of prokaryotes and fungi, respectively. Primers 515F (5′‐GTGCCAGCMGCCGCGGTAA‐3′) and 806R (5′‐GGACTACHVGGGTWTCTAAT‐3′) were used for 16S amplification (Peiffer *et al*., 2013; Guo *et al*., 2016), and primers gITS7 (5′‐GTGARTCATCGARTCTTTG‐3′) and ITS4 (5′‐TCCTCCGCTTATTGATATGC‐3′) for ITS2 amplification (Ihrmark *et al*., 2012; Tedersoo *et al*., 2015). PCR was performed using the HotStarTaq Plus Master Mix Kit (Qiagen, Valencia, CA, USA) with the following settings: an initial 3‐min step at 94°C; 28 cycles of 94°C for 30 s, 53°C for 40 s and 72°C for 1 min; and a final elongation step at 72°C for 5 min. Illumina barcode sequences were then ligated; samples were pooled in equal proportions by molecular weight and pooled PCR products were purified using Agencourt Ampure XP beads (Beckman Coulter, Brea, CA, USA). Illumina libraries were constructed (300 bp paired‐end) and sequenced on the Illumina MiSeq platform following the manufacturer's instructions (Illumina, San Diego, CA, USA).

### Bioinformatic processing

Raw reads were first quality trimmed using a sliding window approach implemented in Trimmomatic v.3 (Bolger *et al*., 2014), with a window size of four base pairs (bp) and a minimum Phred‐scaled quality score threshold of 25. Reads below 150 bp following quality trimming were removed as they were likely to represent incomplete amplicons. Read pairs were then joined using the *join_paired_ends.py* script in the Quantitative Insights in Microbial Ecology (Qiime) package v.1.91 (Caporaso *et al*., 2010b), using a minimum overlap of 50 bp and a maximum percentage difference of two. Paired reads that could not be joined were discarded. Where necessary, joined reads were reverse complemented such that all barcodes were in the 3′ to 5′ orientation. Joined, correctly oriented reads were then demultiplexed using the Qiime package. The accessory script *extract_barcodes.py* was used to separate barcodes from primer sequences for each read, and *split_libraries_fastq.py* was used to sort reads by sample barcode with an additional minimum Phred‐scaled quality score cut‐off of 30. Adaptor and primer sequences were then removed using cutadapt (Martin, 2011). Chimeric sequences were detected with the *identify_chimeric_seqs.py* in Qiime using the Usearch61 algorithm (Edgar, 2010; Edgar *et al*., 2011); these were removed from further analysis.

### Characterization of microbial communities

Operational taxonomic unit (OTU) clustering and classification were performed using the *pick_open_reference_otus.py* workflow in Qiime. Sequences were prefiltered to remove those that shared < 60% identity with the relevant reference databases: GreenGenes (DeSantis *et al*., 2006) for 16S and Unite (Abarenkov *et al*., 2010) for ITS. The remaining sequences were clustered at 97% similarity, and taxonomy was assigned by alignment with PyNAST (Caporaso *et al*., 2010a) to their respective databases. OTUs represented by only a single sequence and 16S OTUs identified as being of mitochondrial or plastid origin were removed from further analysis. Ecological functions were estimated for each OTU using Funguild (Nguyen *et al*., 2016) for fungi and Faprotax (Louca *et al*., 2016) for prokaryotic OTUs.

Dissimilarities between samples (beta diversity) were examined using the *jackknifed_beta_diversity.py* script in Qiime. Samples were repeatedly rarefied to the minimum number of sequences for each dataset over 100 jack‐knifed replicates; beta diversity was calculated using Bray–Curtis dissimilarity (Bray & Curtis, 1957) and clustered using UPGMA (Unweighted Pair Group Method with Arithmetic Mean). Prokaryotic and fungal classes were summarized for each sample using the *summarize_taxa.py* script in Qiime. UPGMA trees and taxonomy barplots at the class level were then visualized using iTol v.3 (Letunic & Bork, 2016). To investigate the effect of species and soil type on microbial communities separately, each dataset (i.e. ITS and 16S) was split into four comparisons: B_root_ vs F_vol‐root_, B_soil_ vs F_vol‐soil_, F_vol‐root_ vs F_cal‐root_ and F_vol‐soil_ vs F_cal‐soil_. The root comparisons, B_root_ vs F_vol‐root_ and F_cal‐root_ vs F_vol‐soil_, were designed to identify species‐ and soil type‐related microbial differences in the roots of the plants. The corresponding soil comparisons, B_soil_ vs F_vol‐soil_ and F_vol‐soil_ vs F_cal‐soil_ were designed to determine whether any such differences were root specific or whether they were simply a product of differences in the surrounding soil. These were further filtered to remove rare OTUs (those occurring in less than three samples) as recommended in the Qiime documentation. For each comparison, Principal Coordinate Analysis (PCoA) was performed using the *jackknifed_beta_diversity.py* script with 100 jack‐knifed replicates rarefied to the minimum per‐sample sequence depth, and PCoAs were based on Bray–Curtis dissimilarity. The significance of the differences within each comparison was assessed using the *compare_categories.py* script in Qiime employing the permutation‐based analysis of variance test (PERMANOVA) with 1000 permutations. To confirm that differential dispersion between test groups was not responsible for significant differences, further permutation‐based tests for homogeneity of dispersion (PERMDISP) were performed using the *compare_categories.py* script with 1000 permutations. To assess within‐sample diversity (alpha diversity) and to determine the proportion of diversity captured by our survey, alpha rarefaction plots were produced for each comparison using the *alpha_rarefaction.py* workflow in Qiime. Alpha diversity was assessed using the Chao1 statistic, a corrected measure of diversity particularly suited to microbial datasets (Chao, 1984; Hughes *et al*., 2016).

To identify the specific OTUs that were differentially abundant between sample categories, we used the *differential_abundance.py* script in Qiime. This uses the DeSeq2 method, which is expected to be more powerful than rarefaction‐based methods (Love *et al*., 2014; McMurdie & Holmes, 2014). The DeSeq2 method includes false discovery rate (FDR) correction for multiple testing. Following on from our initial results, further tests on specific groups were conducted using Fisher's exact tests and Chi‐squared tests with Yate's correction in R: enrichment of arbuscular mycorrhizal fungi (AMF) and nitrogen cycle bacteria amongst significantly differentially abundant OTUs; propensity to be higher or lower in specific sample groups amongst significant OTUs; and differences in the number of AMF OTUs (i.e. species richness) per comparison group. To ensure that our Fisher's exact, PERMANOVA and PERMDISP tests were not affected by OTUs with unassigned taxonomy, which could potentially represent nontarget organisms, we also removed these OTUs and re‐ran all tests.

### Identification of AMF‐related genes under selection in *Howea*

We examined the expression and sequence divergence of AMF‐related genes in *Howea* using transcriptome data from Dunning *et al*. (2016). To do so, genes listed in Mohanta & Bae (2015) as being involved in plant–AMF interactions were identified in Dunning *et al*.'s data, and their measures of expression and sequence differentiation were examined between *Howea* species.

### Seedling survival experiment

To evaluate whether microbes have an effect on *Howea* fitness, we set up a seedling survival experiment on LHI. Two mixes of soil, one volcanic and one calcareous, were made up of soil collected from six sites across LHI (Table S1). Half of each soil mix was then sterilized by passing steam through the soil for 45 min using a horticultural soil steamer. Seeds were collected from two *H. belmoreana* populations and five *H. forsteriana* populations on LHI. Seeds were planted following the standard *Howea* growth protocol at the LHI Nursery (M. Maxwell, Head, pers. comm.). The protocol is designed to maintain humidity and to isolate plants from external sources of microbes. Fifty seeds were planted in each 60 × 80 × 60‐cm^3^ box and using one of the four soil types (i.e. calcareous unsterilized, calcareous sterilized, volcanic unsterilized, volcanic sterilized). Soil was kept moist with 250 ml of water before planting. Seeds were planted to a depth of 20 cm, and then covered with a 3‐cm layer of soil. Each box was placed inside a 1 m × 60 cm clear polyethylene bag, which was then sealed and placed in random order in a shade house with ambient temperature. Bags were not reopened until the end of the experiment. In total, 250 seeds were planted for each seed and soil type combination, resulting in 7000 seeds in 140 bags (Table S2). Bags were then sealed and left at the LHI Nursery for germination and growth over 18 months, following a previous transplant study (Hipperson *et al*., 2016). At the end of the experiment, all bags were opened, and surviving seedlings were counted. Survival on sterilized vs nonsterilized soil was compared for each species–soil combination using a Mann–Whitney *U*‐test in R.

### Visual assessment of AMF colonization

Fixed root samples were rinsed in deionized water to remove traces of fixative. They were cleared by incubation in 10% potassium hydroxide (w/v) at 95°C for 1 h and rinsed twice in deionized water. Cleared samples were bleached in 30% hydrogen peroxide : deionized water (1 : 1, v/v) at room temperature for 30 min to remove residual pigmentation. Samples were then stained in 2% trypan blue : lactic acid : glycerol : deionized water (25 : 300 : 300 : 400, v/v/v/v) at 95°C for 4 min before draining and suspending in glycerol. Stained samples were sectioned longitudinally by hand using a razor blade, and then examined under a compound light microscope. The presence or absence of AMF was assessed for each sample by searching for inter‐ and intracellular aseptate hyphae attached to vesicles, arbuscules and/or hyphal coils. The significance of the differences between the numbers of colonized B_vol_, F_vol_ and F_cal_ trees was assessed using Fisher's exact tests.

### Data and code availability

Raw sequence data were deposited in the National Center for Biotechnology Information Sequence Read Archive (NCBI SRA) database under the project accession PRJNA381758. All custom code is available in Methods S1.

## Results

Across our 32 samples, 16S libraries (prokaryotic communities) contained between 20 812 and 89 324 merged reads, whereas ITS libraries (fungal communities) contained between 26 933 and 190 128 merged reads. A total of 93 097 16S reads and 17 403 ITS reads were removed as suspected chimeras. At a 97% similarity threshold, the total 16S dataset contained 29 648 OTUs, and the ITS dataset contained 7714 OTUs (Tables S3, S4). Of these, 27 982 16S OTUs plus 2990 ITS OTUs were taxonomically assigned to at least the phylum level. Furthermore, 3796 prokaryotic and 1815 fungal OTUs were linked to ecological functions. When plotting increasing numbers of reads against taxonomic richness, the curve did not reach a plateau, suggesting that there remains some additional microbial diversity to sample in the surveyed sites (Fig. S1).

### Total microbial diversity differs between soils rather than between species

**Figure 2:**
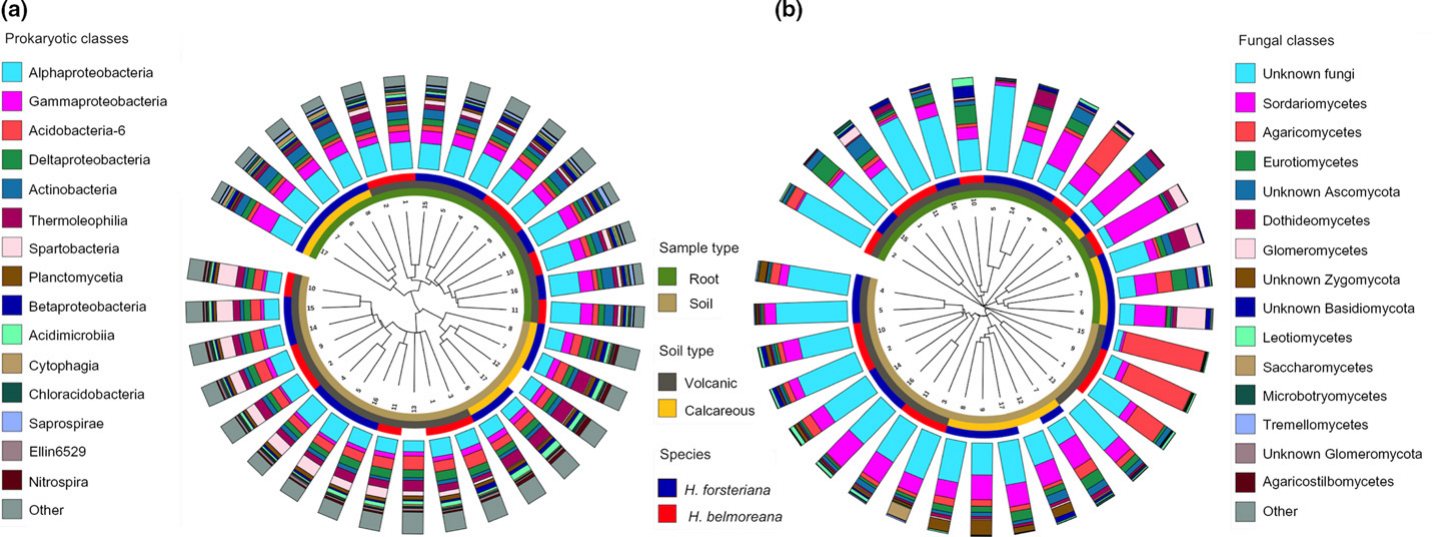
Clustering of samples by (a) prokaryotic 16S and (b) fungal internal transcribed spacer (ITS) abundances. The sample origin and abundances of the 20 most common classes for each are shown in the outer circles; sample names are as in Fig. 1 and Supporting Information Table S1.

**Figure 3:**
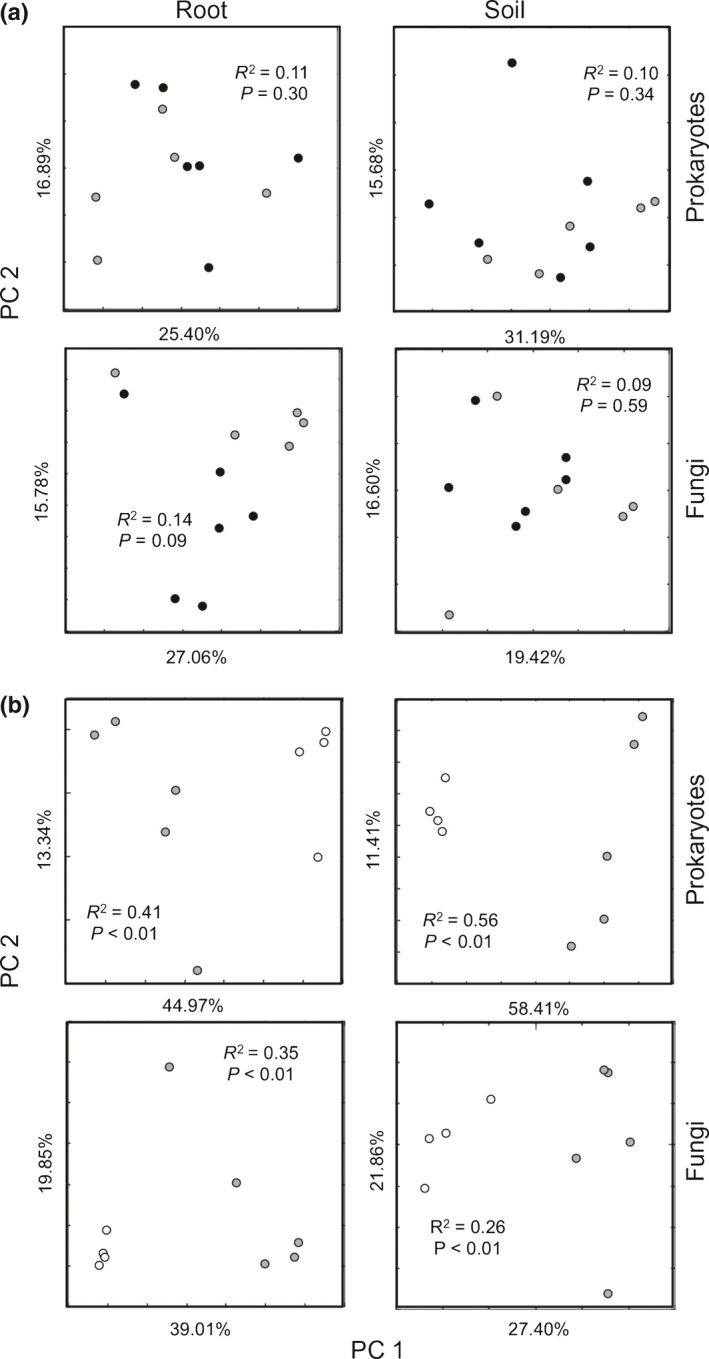
Principal coordinate analysis of metabarcoding data. Comparisons (a) between *Howea forsteriana* and *H. belmoreana* growing on the same soil type (volcanic) and (b) between *H. forsteriana* growing on the two soil types (volcanic and calcareous). For each comparison, plots using both prokaryotic 16S and fungal internal transcribed spacer (ITS) from both root and soil are shown. Colours are as follows: black, *H. belmoreana*; grey, *H. forsteriana* on volcanic soil; white, *H. forsteriana* on calcareous soil. Probabilities and *R* ^2^ values for PERMANOVA tests are shown; note that all comparisons are nonsignificant in (a), but significant in (b) (see text for details).

Clustering of sites by microbial diversity revealed that both prokaryotic and fungal communities clustered primarily by root or soil (Fig. 2). Within root and soil clusters, prokaryotic communities grouped by soil type, whereas fungal communities did not (Fig. 2b). The differences between root and soil were highly significant in both prokaryotic and fungal communities (PERMANOVA, *P *<* *0.001; Table S5). Because *H. forsteriana* grows on both soil types, whereas *H. belmoreana* is restricted to one, we were able to tease apart microbial communities of soil and species without the confounding effect of the other factor. Species‐specific differences in soil and root microbiomes were examined by comparing B_root_ vs F_vol‐root_. To determine whether any observed difference was simply a result of differences in the surrounding soil, B_soil_ and F_vol‐soil_ were also compared. Neither of these comparisons was significant (Fig. 3a; Table S5). To examine soil type‐specific differences, we compared F_vol‐root_ vs F_cal‐root_ and F_vol‐soil_ vs F_cal‐soil_ using PERMANOVA tests. This revealed significant differences for prokaryotic (F_vol‐root_ vs F_cal‐root_: *R* ^2^ = 0.41, *P* < 0.001; F_vol‐soil_ vs F_cal‐soil_: *R* ^2^ **= 0.56, *P *<* *0.001) and fungal (F_vol‐root_ vs F_cal‐root_: *R* ^2^ **= 0.35, *P *<* *0.001; F_vol‐soil_ vs F_cal‐soil_: *R* ^2^ **= 0.26, *P *<* *0.001) communities in both root and soil samples (Fig. 3b; Table S5). PERMDISP tests revealed no significant differences in dispersal between samples; therefore, we can be confident in the PERMANOVA results (Table S5). When we re‐ran PERMANOVA and PERMDISP tests with unassigned taxa removed, there were no differences in significance/nonsignificance of tests, suggesting that significant differences were not driven by unknown OTUs (Table S5).

### Multiple OTUs are differentially abundant between comparison groups

**Figure 4:**
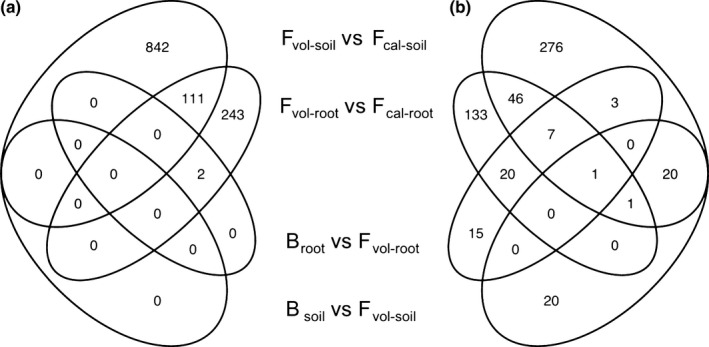
Venn diagrams showing the numbers and overlap of significantly differentially abundant prokaryotic 16S (a) and fungal Internal Transcribed Spacer (b) operational taxonomic units (OTUs) between comparisons. B_root_, root samples from *Howea belmoreana* growing on volcanic soil; B_soil_, soil samples from *H. belmoreana* growing on volcanic soil; F_cal‐root_, root samples from *H. forsteriana* growing on calcareous soil; F_cal‐soil_, soil samples from *H. forsteriana* growing on calcareous soil; F_vol‐root_, root samples from *H. forsteriana* growing on volcanic soil; F_vol‐soil_, soil samples from *H. forsteriana* growing on volcanic soil.

We then examined relative read numbers as a proxy for the differential abundance of individual OTUs in B_root_ vs F_vol‐root_, B_soil_ vs F_vol‐soil_, F_vol‐root_ vs F_cal‐root_ and F_vol‐soil_ vs F_cal‐soil_. Following correction for multiple comparisons, the numbers of significantly differentially abundant OTUs per comparison varied widely from 0 to 953 (Fig. 4; Tables 1, S3, S4). Amongst prokaryotic communities, all significantly differentially abundant OTUs were associated with soil type (Fig. 4a). This included 953 differentially abundant OTUs between F_vol‐soil_ and F_cal‐soil_ (417 were more abundant in F_cal‐soil_ and 536 were more abundant in F_vol‐soil_; Table 1) and 356 differentially abundant OTUs between F_vol‐root_ and F_cal‐root_ (178 were more abundant in F_cal‐root_ and 178 were more abundant in F_vol‐root_; Table 1). Of these, 111 OTUs were differentially abundant between both the F_vol‐soil_ vs F_cal‐soil_ and F_vol‐root_ vs F_cal‐root_ comparisons (Fig. 4a). Two of the differentially abundant OTUs between F_vol‐root_ and F_cal‐root_ were also differentially abundant between B_root_ and F_vol‐root_, but no other species‐specific differences in prokaryotic communities were found. In the fungal dataset, the comparisons of soil types again contained the highest numbers of significantly differentially abundant OTUs (Fig. 4b). A total of 354 OTUs were differentially abundant between F_vol‐soil_ and F_cal‐soil_ (186 were more abundant in F_cal‐soil_ and 168 were more abundant in F_vol‐soil_; Table 1) and 208 were differentially abundant between F_vol‐root_ and F_cal‐root_ (126 were more abundant in F_cal‐root_ and 82 were more abundant in F_vol‐root_; Table 1). Unlike in prokaryotic communities, however, the fungal dataset also contained multiple OTUs that were significantly differentially abundant in the species comparisons. Forty‐six fungal OTUs were significantly differentially abundant between the roots of the two species on volcanic soil (27 were more abundant in B_root_ and 19 were more abundant in F_vol‐root_; Table 1). Although 42 fungal OTUs were also differentially abundant between B_soil_ and F_vol‐soil_ (11 were more abundant in B_soil_ and 31 were more abundant in F_vol‐soil_; Table 1), there was only one OTU in both of these sets (Fig. 4b). This indicates that species‐specific differences in root‐associated fungal OTUs were not simply the product of differences in the surrounding soils.

**Table 1:** Significantly differentially abundant operational taxonomic units (OTUs) between each comparison

Comparison	Highest abundance	Prokaryotes	Fungi
B_soil_ vs. F_vol‐soil_	B_soil_	0	11
	F_vol‐soil_	0	31
B_root_ vs. F_vol‐root_	B_root_	0	27
	F_vol‐root_	2	19
F_vol‐soil_ vs. F_cal‐soil_	F_cal‐soil_	417	186
	F_vol‐soil_	536	168
F_vol‐root_ vs. F_cal‐root_	F_cal‐root_	178	126
	F_vol‐root_	178	82

B_root_, root samples from *Howea belmoreana* growing on volcanic soil; B_soil_, soil samples from *H. belmoreana* growing on volcanic soil; F_cal‐root_, root samples from *H. forsteriana* growing on calcareous soil; F_cal‐soil_, soil samples from *H. forsteriana* growing on calcareous soil; F_vol‐root_, root samples from *H. forsteriana* growing on volcanic soil; F_vol‐soil_, soil samples from *H. forsteriana* growing on volcanic soil.

### Arbuscular mycorrhizal fungi are depleted in *H. forsteriana* on volcanic soil

Strikingly, fungal OTUs that were significantly differentially abundant in root tissue were dominated by AMF. Of the 16 differentially abundant OTUs between B_root_ and F_vol‐root_ that could be identified beyond the kingdom level (Table S4), half were assigned to the phylum Glomeromycota (Glomeromycota has recently been downgraded to Glomeromycotina, but, as this is not yet reflected in the Unite database, we refer to them as Glomeromycota here; Spatafora *et al*., 2016). All but one Glomeromycota species are AMF; hence, we use both terms synonymously here. The greater probability of AMF OTUs to be differentially abundant between B_root_ and F_vol‐root_ relative to other taxa was significant (Fisher's exact test: *P *=* *0.049; Table S6). Similarly, of the 89 differentially abundant OTUs between F_cal‐root_ and F_vol‐root_ that could be identified beyond the kingdom level (Table S4), 44 were AMFs. Again, the greater probability of AMF OTUs to be differentially abundant between F_cal‐root_ and F_vol‐root_ was highly significant (Fisher's exact test; *P *<* *0.001; Table S7). None of the differentially abundant AMF OTUs in the B_root_ vs F_vol‐root_ comparison, and only eight of the differentially abundant AMF OTUs in the F_cal‐root_ vs F_vol‐root_ comparison, were also differentially abundant in their corresponding soil tests (B_soil_ vs F_vol‐soil_; F_cal‐soil_ vs F_vol‐soil_). This indicates that these differences were root‐specific rather than being driven by differences in the AMFs of surrounding soils (Table S4).

**Figure 5:**
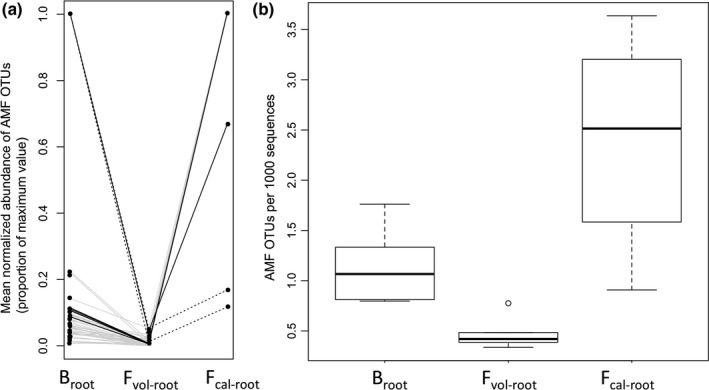
Reduced abundance and species richness of arbuscular mycorrhizal fungi (AMF) in the roots of *Howea forsteriana* on volcanic soil. (a) Mean normalized abundance (normalized by reads per sample; expressed as a proportion of the maximum value per operational taxonomic unit, OTU) for each differentially abundant AMF OTU. Each set of three points connected by a line represents a single OTU. Points connected by solid grey lines are OTUs differentially abundant between *H. forsteriana* roots in calcareous vs volcanic soil, points connected by dotted black lines are OTUs differentially abundant between *H. belmoreana* vs *H. forsteriana* roots on volcanic soil, and points connected by solid black lines are OTUs significantly differentially abundant in both comparisons. Lines are only included to show the correspondence of points between datasets and do not represent trend lines. (b) Boxplots of the number of AMF OTUs. B_root_, *H. belmoreana* roots on volcanic soil; F_vol‐root_, *H. forsteriana* roots on volcanic soil; F_cal‐root_, *H. forsteriana* roots on calcareous soil. Boxes show interquartile ranges (IQR), bold horizontal lines show medians, whiskers show ranges excluding outliers and circles show outliers (defined as being outside the IQR by over 1.5 × IQR).

Differentially abundant AMF OTUs between B_root_ vs F_vol‐root_ and F_vol‐root_ vs F_cal‐root_ were always lower in F_vol‐root_ (Fig. 5a). This is in striking contrast with nonmycorrhizal differentially abundant OTUs, which were significantly higher or lower in F_vol‐root_ in equal proportions (Tables S8, S9). The higher probability of significantly differentially abundant AMF OTUs to be lower in abundance in F_vol‐root_ relative to other differentially abundant OTUs was significant in both comparisons (Fisher's exact tests: B_root_ vs F_vol‐root_: *P *=* *0.014; F_vol‐root_ vs F_cal‐root_: *P *<* *0.001). In addition to a difference in AMF abundance, there was a significant reduction in AMF species richness in F_vol‐root_ relative to both B_root_ and F_cal‐root_ (Mann–Whitney *U*‐tests comparing the number of AMF OTUs scaled by library size: B_root_ vs F_vol‐root_: W = 30, *P *=* *0.004; F_vol‐root_ vs F_cal‐root_: W = 20, *P *=* *0.016; Fig. 5b). When we removed all the OTUs that were not assigned to any taxa, all previous patterns of AMF abundance remained significant, with the exception of the test shown in Table S8. However, this test had very low numbers of OTUs, and so it may have suffered from a lack of power. It is possible that F_vol‐root_ may have contained a large number of rare AMF OTUs, which would have been removed by our filter for sequences occurring in less than three samples. To evaluate this, we also examined the low‐abundance OTUs removed from this analysis. These displayed the same pattern as the main dataset, with AMF OTUs having lower abundance and species richness in F_vol‐root_ than in either F_cal‐root_ or B_root_ (Table S10).

Our visual examination of AMF colonization supported the conclusions of the metabarcoding analysis, that is, AMF colonization was lower in F_vol‐root_ than in either B_root_ or F_cal‐root_ (Fig. S2; Table S11). There was no significant correlation between total AMF abundance from the metabarcoding analysis and the percentage of samples with AMF colonization per site, but the difference in colonization between F_vol‐root_ and B_root_ was significant (Fisher's exact test: *P *=* *0.049; Table S12).

To gain insight into the possible causes of differences in AMF abundance between groups, we identified 19 potential mycorrhizal‐associated genes in the *Howea* reference transcriptome following Mohanta & Bae (2015) (Table S13). Ten of the genes were differentially expressed between *H. forsteriana* and *H. belmoreana*, although this differential expression was in leaf or floral tissue rather than root, and none were differentially expressed between *H. forsteriana* on calcareous vs volcanic soil. However, both *Howea* homologues of the strigolactone biogenesis protein *CCD8a* had a high level of sequence differentiation between *H. belmoreana* and *H. forsteriana* (F_ST_ = 0.8 and F_ST_ = 1). The same was found for the ammonium transporter *AMT2* (F_ST_ = 1). These genes may have a role in AM differences (Mohanta & Bae, 2015).

### Variation in abundance of pathogens and ecologically important microbes

In addition to the clear differences in AM fungi between comparison groups, several other OTUs were differentially abundant with potential relevance to plant fitness. Amongst non‐AMF fungal OTUs significantly differentially abundant between B_root_ and F_vol‐root_, only three were assigned ecological functions, including a known plant pathogen, *Ilyonectria macrodidyma*, which was significantly more abundant in *H. forsteriana* roots. Non‐AMF differentially abundant fungal OTUs between F_vol‐root_ and F_cal‐root_ also included four known plant pathogens. Two of these were in the genus *Cylindrocarpon*, one was *Rhizopycnis vagum* and one was *Fusarium oxysporum*. All four were more abundant in the roots of *H. forsteriana* on calcareous soil. In addition, one OTU from the genus *Chaetomium* had significantly higher read abundance in the roots of *H. forsteriana* on calcarenite soil; some members of *Chaetomium* are known to increase the germination rate in *Cecropia* trees (Gallery *et al*., 2007).

Amongst prokaryotic communities, there was an excess of nitrogen cycle bacteria in F_vol‐root_ relative to F_cal‐root_. Differentially abundant prokaryotic OTUs between F_vol‐root_ and F_cal‐root_ were significantly enriched for bacteria whose ecological functions are related to the nitrogen cycle (i.e. anaerobic ammonia oxidation, aerobic ammonia oxidation, anaerobic nitrite oxidation, aerobic nitrite oxidation, nitrate ammonification, nitrate denitrification, nitrate reduction, nitrate respiration, nitrification, nitrite ammonification, nitrite denitrification, nitrite respiration, nitrogen fixation, nitrogen respiration, nitrous oxide denitrification; Chi‐squared test with Yate's correction: χ^2^ = 14.80, *P *<* *0.001), perhaps reflecting the higher nitrogen levels in volcanic soil.

### Soil sterilization reduces seedling success in all soil–species combinations except for *H. forsteriana* grown on volcanic soil

**Figure 6:**
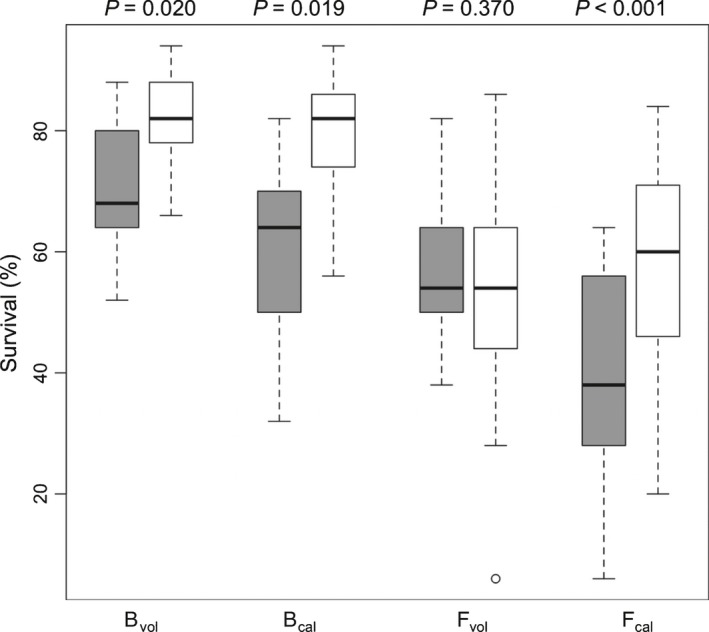
The effect of soil sterilization on seed germination and survival in *Howea forsteriana* and *H. belmoreana* grown on volcanic and calcareous soil. Boxplots show the proportion of seedling survival in sterilized (grey) and unsterilized (white) soil. *P* values from Mann–Whitney *U*‐tests are shown above each pairwise comparison; B_vol_, *H. belmoreana* growing on volcanic soil; B_cal_, *H. belmoreana* growing on calcareous soil; F_vol_, *H. forsteriana* growing on volcanic soil; F_cal_, *H. forsteriana* growing on calcareous soil. Boxes show interquartile ranges (IQR), bold horizontal lines show medians, whiskers show ranges excluding outliers and circles show outliers (defined as being outside the IQR by over 1.5 × IQR).

To evaluate the effect of microbes on *Howea* fitness, we conducted a field experiment in which seedling survival was compared on sterilized vs unsterilized soils. Seedling survival per replicate ranged from 0 to 94% (Table S13). In *H. belmoreana*, sterilization of both soil types resulted in a significant decrease in seedling survival (Fig. 6). In *H. forsteriana*, however, although sterilization of calcareous soil resulted in a strong decrease in survival, sterilization of volcanic soil had no effect, in line with the low read abundance and richness of plant‐beneficial fungal OTUs in *H. forsteriana* on volcanic soil (see above). This suggests that, although the microbiome has a net beneficial effect on seedlings in *H. belmoreana* on both soil types, and in *H. forsteriana* on calcareous soil, no such beneficial effect exists in *H. forsteriana* on volcanic soil.

## Discussion

All species are affected by interspecific interactions, and those between multicellular organisms and the microbiomes they support may be especially important. Microbes are known to promote speciation (Brucker & Bordenstein, 2013), coexistence (Waterman *et al*., 2011) and extinction (Smith *et al*., 2006) of their hosts, yet they are not commonly studied as agents of evolutionary change in plants and animals. In this study, we uncovered a link between microbial diversity and adaptation and coexistence in *Howea* palms, a prominent example of sympatric speciation (Savolainen *et al*., 2006).

Arbuscular mycorrhizal fungi appear to be significantly depleted in the roots of *H. forsteriana* on volcanic soil, relative to both *H. forsteriana* on calcareous soil and *H. belmoreana*. This manifests as both a decrease in richness of AMF OTUs and a decrease in their abundance. Although the ITS2 region is commonly used for the assessment of fungal species richness and abundance, it is necessary to acknowledge some caveats associated with this approach. Fungal abundance is difficult to estimate from ITS metabarcoding data owing to differences in ribosomal copy number between species, PCR biases and differences in DNA extraction efficacy (Taylor *et al*., 2016). Therefore, it is possible that some of the differential abundance we observed was a result of these factors. However, there is no reason to suspect that these would lead to a systematic bias in the current study. Furthermore, the primer combination used in this study has been shown to have the best accuracy in the assessment of fungal abundance relative to other ITS primer combinations (Ihrmark *et al*., 2012; Taylor *et al*., 2016); therefore, it is likely that our results broadly reflect actual abundance, although we cannot rule out some effect of, for example, fungal lineage‐specific copy number variation.

**Figure 7:**
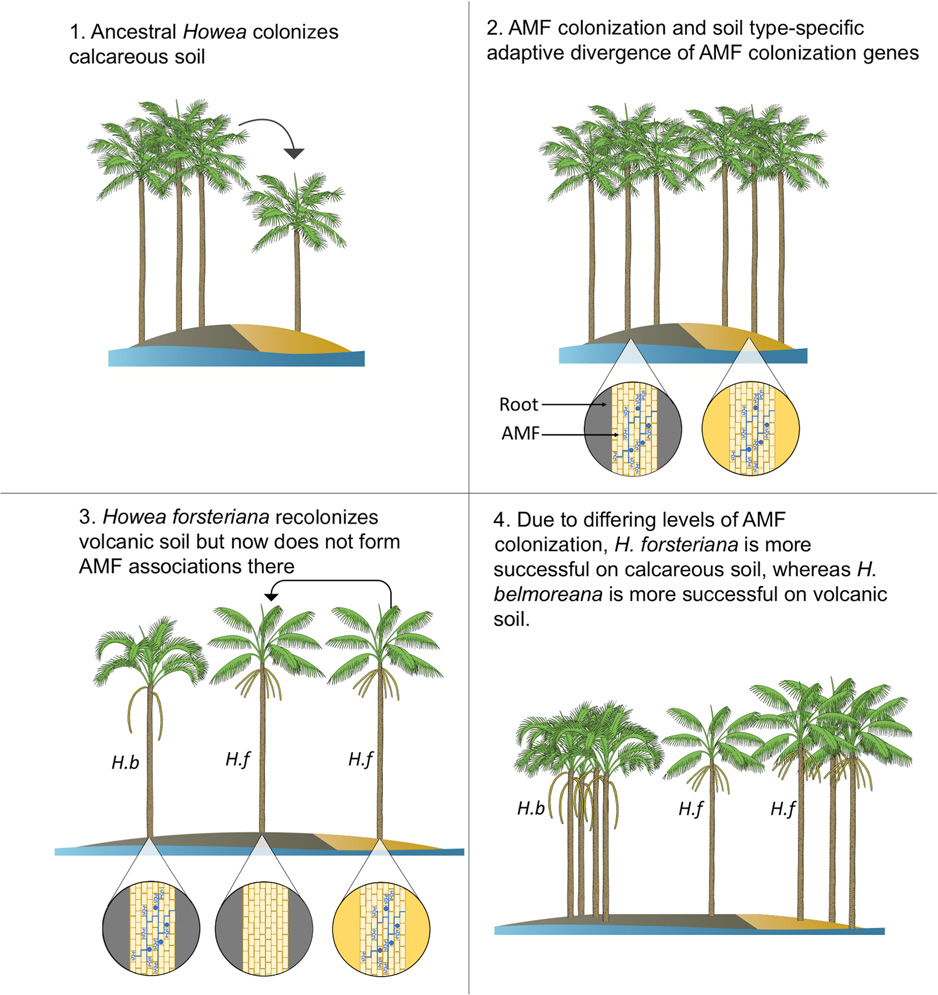
One possible scenario leading to the reduction of arbuscular mycorrhizal fungi (AMF) in *Howea forsteriana* (*H.f*) on volcanic soil relative to both *H. forsteriana* on calcareous soil and to *H. belmoreana* (*H.b*).

The symbiosis between AMF and land plants probably played a key role in their success and is present in 80% of plant species today (Smith & Read, 1997; Brundrett, 2002; Field *et al*., 2015). In exchange for carbohydrates, the fungi provide the associated plant with resources such as phosphate and nitrogen. The presence or absence of AMF is therefore probably key to plant fitness, including in palms (Moora *et al*., 2011; Al‐Karaki, 2013). Thus, the reduction in AMF in *H. forsteriana* on volcanic soil may place it at a substantial disadvantage compared with *H. belmoreana*. Plant RNA sequence data from a previous study (Dunning *et al*., 2016) have indicated that adaptive divergence between *Howea* species may be linked to the difference in AMF abundance uncovered here. Specifically, mycorrhizal‐associated genes show signs of adaptive divergence between *H. forsteriana* and *H. belmoreana*. Initiation of AMF symbiosis is driven by strigolactones released by plant roots (Akiyama *et al*., 2005). The *CCD8a* gene is a key part of the strigolactone biogenesis pathway (Gomez‐Roldan *et al*., 2008) and shows molecular signatures of divergent selection between *H. forsteriana* and *H. belmoreana*. A second gene, *AMT2*, also showed high levels of differentiation. This is involved in ammonium transport and is specific to mycorrhizal associations (Guether *et al*., 2009). Overall, the sequence data bolster the view that genetic divergence between *Howea* species has affected their ability to form AMF associations. Such a situation could have come about via a number of routes. One plausible hypothesis (illustrated in Fig. 7) is that, following its initial colonization of calcareous soil, the ancestor of *H. forsteriana* underwent adaptation to optimize its interactions with AMF in the calcareous soil environment. This could have taken the form, for example, of a change in the threshold of chemical triggers (e.g. phosphate starvation) needed for the production of the root exudates that trigger AMF colonization. Following re‐colonization of volcanic soil, *H. forsteriana* may then have been poorly suited to forming AMF interactions on volcanic soil.

If the mycorrhizal shortfall in *H. forsteriana* on volcanic soil was of adaptive significance, we would expect sterilization of volcanic soil to have a less damaging effect on *H. forsteriana* than on *H. belmoreana*. Similarly, we may expect the sterilization of calcareous soil to be more deleterious than the sterilization of volcanic soil for *H. forsteriana*. This was precisely the pattern we observed. Soil sterilization caused a significant decrease in seedling survival in *H. belmoreana* on both soil types and in *H. forsteriana* on calcareous soil, indicating that they receive a net benefit from soil microbiota and that plant‐beneficial microbes are being removed via sterilization. Conversely, the lack of an effect in *H. forsteriana* on volcanic soil suggests that there is no net benefit of the native volcanic microbiome for *H. forsteriana*. Thus, our results indicate that *H. forsteriana* only benefits from the native microbiome of calcarenite soil, whereas *H. belmoreana* is able to benefit from that of volcanic soil. Although *H. belmoreana* was also able to benefit from the microbiome of calcareous soil in our experiment, and indeed showed a higher survival rate than *H. forsteriana* on calcareous soil, it does not survive to adulthood on calcareous soil, and so this result is unlikely to be of ecological importance in the wild. Here, we only measured one aspect of evolutionary fitness, that is, seedling survival. It is likely that there is strong selection against *H. belmoreana* on calcareous soil at a later stage in the lifecycle or in certain conditions, such as during drought. Furthermore, the specific microbial taxa responsible for these fitness differences could not be identified. Future experiments would benefit from the inoculation of soil with specific native microbial taxa, examination of the mycorrhizal distribution and function, experimental cross‐inoculation of sterile soil with microbiomes of calcareous vs volcanic soils, examination of multiple fitness measures and the use of plants at different life stages.

Although the experimental treatment of plants with isolated microbial taxa would be required to confirm the taxa responsible for these beneficial effects, AMF appear to be the best candidates based on our results. AMF were significantly more likely than other fungal OTUs to be differentially abundant between *H. forsteriana* and *H. belmoreana* on volcanic soil, and are expected to have beneficial effects on their plant hosts *a priori*. Nevertheless, many other taxa could potentially be involved. For example, several known bacterial and fungal plant pathogens were differentially abundant in the comparison between *H. forsteriana* in calcareous and volcanic soil. Furthermore, many microbial taxa are likely to have unknown interactions with their plant hosts. This is illustrated by one of our significantly differentially abundant fungal OTUs, a *Chaetomium* species. Although *Chaetomium* are primarily known as saprotrophs (and were annotated as such in our Funguild analysis), their presence is associated with higher germination rates in some tropical tree species via an unknown mechanism (Gallery *et al*., 2007). This underlines the fact that much of the microbial world is of unknown ecological function and, at present, many of the complex interspecific interactions likely to be present in the soil can only be guessed at. Furthermore, we document here the microbial diversity of LHI, a UNESCO‐designated world heritage site of conservation concern. A large portion of the microbes uncovered were known pathogens (3.73% of fungal OTUs and 0.11% of prokaryotic OTUs; Tables S2, S3; Fig. S3), with potentially harmful effects to LHI's threatened flora and fauna, a situation which may need attention in the future.

We found that total microbial diversity differed by soil type, but not by *Howea* species. This was matched by a substantially greater number of differentially abundant OTUs between soil types than between species. Previous studies have indicated that both soil type and plant species can affect the microbial composition of soils (Marschner *et al*., 2001; Wieland *et al*., 2001; Buyer *et al*., 2002; Girvan *et al*., 2003; Chen *et al*., 2007). Although some studies found that plant species had a greater effect (Wieland *et al*., 2001), others found a stronger effect for soil type (Buyer *et al*., 2002; Girvan *et al*., 2003). It is likely that the magnitude of chemical and structural differences between soil types determines to what extent they affect microbial communities. Calcareous and volcanic soils are extremely different in terms of pH, chemical composition and structure, and this appears to result in a major effect on microbial diversity on LHI.

In addition to affecting their adaptation and promoting coexistence, it is possible that the respective microbiomes of the two *Howea* species could have been involved in their speciation. A plausible mechanism for this could be via an effect on flowering time, and there are two distinct mechanisms by which this could occur. First, a microbiome‐mediated plastic shift in flowering time could have led to the initial isolation of populations on different soil types. Second, adaptation to differing microbiomes could have led to a heritable, pleiotropic shift in flowering time. Although many studies have reported a microbial impact on flowering time, including several which have implicated AMF (Gaur *et al*., 2000; Korves & Bergelson, 2003; Salvioli *et al*., 2012; Wagner *et al*., 2014; Jin *et al*., 2015; Lyons *et al*., 2015; Panke‐Buisse *et al*., 2015), they did not document a genetic explanation for their observations. Because several genes are known to be involved in both pathogen defence and flowering time (Kidd *et al*., 2009; Lai *et al*., 2014; Kazan & Lyons, 2016), we speculate that adaptation of these genes in response to soil microbes could plausibly cause a shift in flowering time, including in *Howea*.

We argue that the role of the microbiome in evolutionary change has been underestimated. Several examples of microbe‐mediated host evolution have been uncovered in animals, such as hybrid lethality as a result of gut microbes in wasps (Brucker & Bordenstein, 2013), mating preference shifts as a result of commensal bacteria in fruit flies (Sharon *et al*., 2013) and the profound and wide‐ranging evolutionary consequences of *Wolbachia* on their invertebrate hosts (Rokas, 2000). Far fewer examples exist in plants, but, given the universality of plant–microbe associations, and their known potential for the promotion of evolutionarily important effects, such as flowering time shifts and increased invasiveness, the results presented here may represent a situation more common in plants than currently appreciated. For example, in sympatric orchids in South Africa, mycorrhizae allowed their coexistence, whereas pollinators drove their divergence (Waterman *et al*., 2011). In *Howea*, without the benefit of mycorrhizae, *H. belmoreana* may have been outcompeted by *H. forsteriana*, and therefore gone extinct. Also, given the key role of soil in the scenario for sympatric speciation in *Howea*, it is possible that microbes also had an important role in species divergence via pleiotropic genes, which would have had an effect on both soil adaptation (here via microbial interactions) and flowering time, as suggested by Dunning *et al*. (2016). Overall, our results provide the first evidence of a significant role for microbes in the evolutionary ecology of *Howea*, a situation which may prove to be common in plants.

## Author contributions

V.S., R.D‐K., M.I.B. and O.G.O. designed the experiments; V.S. supervised the research; O.G.O., R.D‐K. and I.H. conducted the fieldwork; O.G.O., M.I.B. and R.D‐K. conducted the laboratory work; R.D‐K. conducted some preliminary analysis; O.G.O. conducted data analysis and drafted the initial manuscript; W.J.B. and C.G.N.T. advised on methods; V.S. and O.G.O. finalized the manuscript with contributions from all co‐authors.

## Supporting information

Please note: Wiley Blackwell are not responsible for the content or functionality of any Supporting Information supplied by the authors. Any queries (other than missing material) should be directed to the *New Phytologist* Central Office.

**Fig. S1** Alpha rarefaction plots for each comparison tested.
**Fig. S2** Microscopic validation of metabarcoding‐based arbuscular mycorrhizal fungal abundance estimates.
**Fig. S3** Pie charts showing the ecological guilds of all known fungal and prokaryotic pathogens identified.
**Methods S1** Custom bash code used during sequence data analysis.

**Table S1** Location and sample information for all metabarcoding samples
**Table S2** Survival rate on sterilized vs nonsterilized soil in *Howea belmoreana* and *Howea forsteriana*
**Table S3** Summary of 16S operational taxonomic units (OTUs)
**Table S4** Summary of ITS operational taxonomic units (OTUs)
**Table S5** PERMANOVA and PERMDISP tests between sample groupings
**Table S6** Fisher's exact test of excess of arbuscular mycorrhizal fungi (AMF) in differentially abundant operational taxonomic units (OTUs) between *Howea belmoreana* and *H. forsteriana* on volcanic soil
**Table S7** Fisher's exact test of excess of arbuscular mycorrhizal fungi (AMF) in differentially abundant operational taxonomic units (OTUs) between volcanic and calcareous soil in *H. forsteriana*
**Table S8** Fisher's exact test of higher or lower abundance in *Howea belmoreana* relative to *H. forsteriana* amongst differentially abundant arbuscular mycorrhizal fungi (AMF) operational taxonomic units (OTUs) relative to non‐AMF differentially abundant ITS OTUs
**Table S9** Fisher's exact test of higher or lower abundance in calcareous soil relative to volcanic soil amongst differentially abundant arbuscular mycorrhizal fungi (AMF) operational taxonomic units (OTUs) relative to non‐AMF differentially abundant ITS OTUs
**Table S10** Arbuscular mycorrhizal fungal abundance and species richness in discarded low‐abundance samples
**Table S11** Visual assessment of arbuscular mycorrhizal fungal colonization in five trees per sample site
**Table S12** Fisher's exact test of differences in the presence of visible arbuscular mycorrhizal fungi between comparison groups
**Table S13** Expression and sequence divergence of mycorrhizal‐associated genes in the *Howea* reference transcriptome
