# Supplementary material for: Arbuscular mycorrhizal fungi promote coexistence and niche divergence of sympatric palm species on a remote oceanic island
Source: New Phytol. 2017 Oct 16;217(3):1254–66. doi: 10.1111/nph.14850 (PMC5813143; doi:10.1111/nph.14850)
Supplement: Supplementary file 1 — Fig. S1 Alpha rarefaction plots for each comparison tested. Fig. S2 Microscopic validation of metabarcoding‐based arbuscular mycorrhizal fungal abundance estimates. Fig. S3 Pie charts showing the ecological guilds of all known fungal and prokaryotic pathogens identified. Methods S1 Custom bash code used during sequence data analysis. [file NPH-217-1254-s001.pdf]

***New Phytologist* Supporting Information**

Article title Arbuscular mycorrhizal fungi promote coexistence and niche divergence of sympatric palm species on a remote oceanic island

Authors: Owen G. Osborne, Rishi De-Kayne, Martin I. Bidartondo, Ian Hutton, William J. Baker, Colin G. N. Turnbull, Vincent Savolainen

Article acceptance date: 14 September 2017

The following Supporting Information is available for this article:

Fig. S1 Alpha rarefaction plots for each comparison tested. Lines are shown for every sample within each comparison.

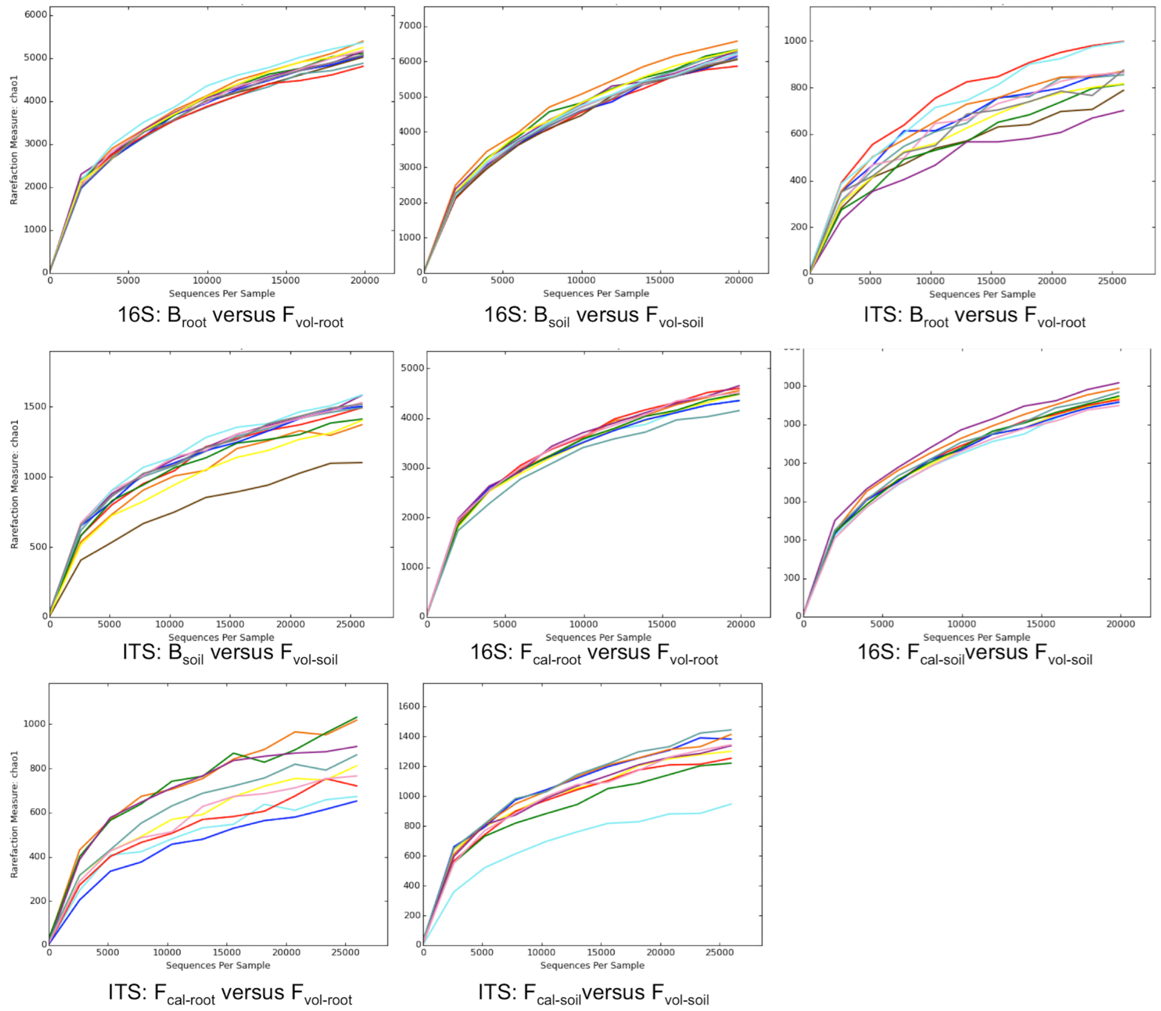

Fig. S2 Microscopic validation of metabarcoding-based arbuscular mycorrhizal fungi abundance estimates. (a) *H. forsteriana* growing on volcanic soil (F_{vol}) had a lower percentage of AM-colonised trees per site than either *H. belmoreana* (B_{vol}) or *H. forsteriana* growing on calcareous soil (F_{cal}). Bars show mean percentage of trees with AM colonisation per site and error bars shown Standard Error of the Mean (SEM). (b-d) typical AM structures found in our samples. Arbuscules (A), vesicles (V) and hyphal coils (C) are marked with arrows. Horizontal bars are 50um scale bars. Example micrographs are from samples 10A (b,d) and 9D (c).

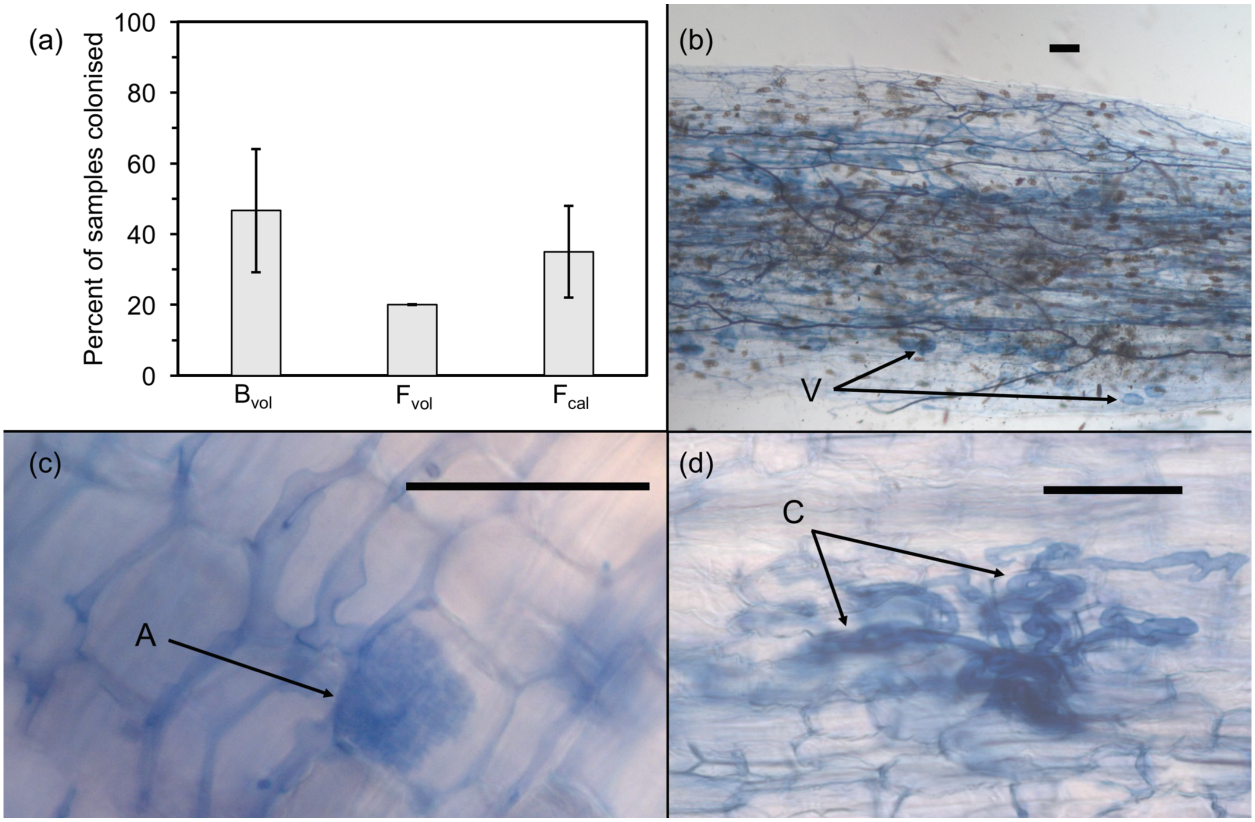

Fig. S3 Pie charts showing the ecological guilds of all known fungal (a) and prokaryotic (b) pathogens identified**.**

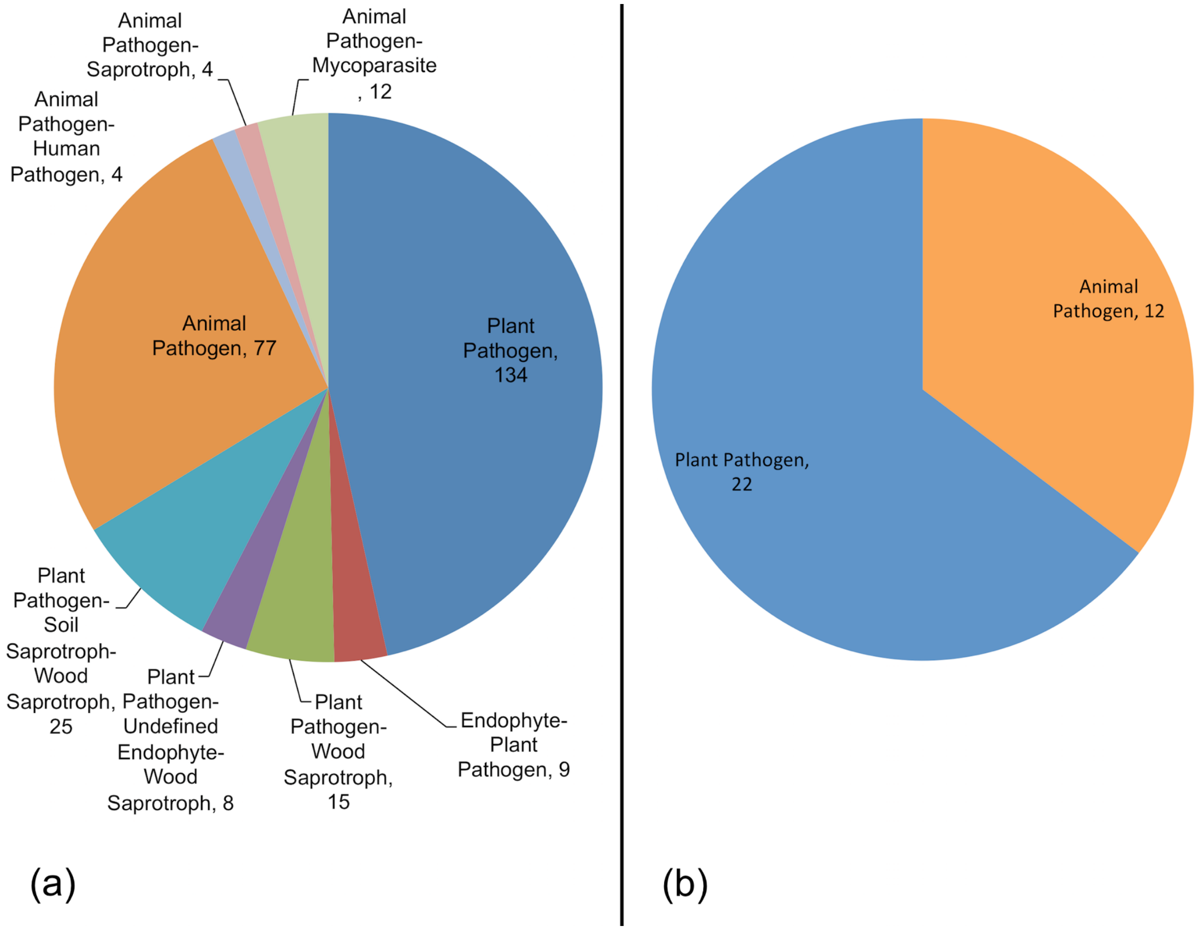

Table S1 Location and sample information for all metabarcoding samples (supplementary Excel spreadsheet).

Table S2 Survival rate on sterilised versus non-sterilised soil in H. belmoreana and H. forsteriana (supplementary Excel spreadsheet).

Table S3 Summary of 16S OTUs (supplementary Excel spreadsheet).

Table S4 Summary of ITS OTUs (supplementary Excel spreadsheet).

Table S5 PERMANOVA and PERMDISP tests between sample groupings (supplementary Excel spreadsheet).

Table S6 Fisher's exact test of excess of AMF in differentially abundant OTUs between H. belmoreana and H. forsteriana on volcanic soil PERMANOVA and PERMDISP tests between sample groupings (supplementary Excel spreadsheet).

Table S7 Fisher's exact test of excess of AMF in differentially abundant OTUs between volcanic and calcareous soil in H. forsteriana (supplementary Excel spreadsheet).

Table S8 Fisher's exact test of higher or lower abundance in H. belmoreana relative to H. forsteriana among differentially abundant AMF OTUs relative to non-AMF differentially abundant ITS OTUs (supplementary Excel spreadsheet).

Table S9 Fisher's exact test of higher or lower abundance in calcareous soil relative to volcanic soil among differentially abundant AMF OTUs relative to non-AMF differentially abundant ITS OTUs (supplementary Excel spreadsheet).

Table S10 AMF abundance and species richness in discarded low abundance samples (supplementary Excel spreadsheet).

Table S11 Visual assessment of AMF colonisation in five trees per sample site. (supplementary Excel spreadsheet).

Table S12 Fisher's exact test of differences in presence of visible AMF between comparison groups (supplementary Excel spreadsheet).

Table S13 Expression and sequence divergence of mycorrhizal associated genes in the Howea reference transcriptome (supplementary Excel spreadsheet).

Methods S1 The following custom code was used during sequence data analysis:

Bash code used to split forward and backward oriented reads (following paired end merging)

```
#16S
grep -B 1 -A 2  "^GA......GTGCCAGC.GCCGCGGTAA" ./16S/Q25_J50_P2/fastqjoin.join.fastq |
sed '/^--$/d' > ./16S/16S_joined_forward_or.fastq
grep -B 1 -A 2  "TTACCGCGGC.GCTGGCAC......TC$" ./16S/Q25_J50_P2/fastqjoin.join.fastq |
sed '/^--$/d' > ./16S/16S_joined_reverse_or.fastq

#ITS
grep -B 1 -A 2  "^TC......GTGA.TCATCGAATCTTTG" ./ITS/Q25_J50_P2/fastqjoin.join.fastq |
sed '/^--$/d' > ./ITS/ITS_joined_forward_or.fastq
grep -B 1 -A 2  "CAAAGATTCGATGA.TCAC......GA$" ./ITS/Q25_J50_P2/fastqjoin.join.fastq |
sed '/^--$/d' > ./ITS/ITS_joined_reverse_or.fastq
```

Bash code used to reorient backwards reads and re-concatenate fastq files (requires seqtk)

```
#16S
seqtk seq -r ./16S/16S_joined_reverse_or.fastq > ./16S/16S_joined_reverse_or_revcomp.fastq
cat ./16S/16S_joined_forward_or.fastq ./16S/16S_joined_reverse_or_revcomp.fastq >
./16S/16S_joined_all_oriented.fastq

#ITS
```

```
seqtk seq -r ./ITS/ITS_joined_reverse_or.fastq > ./ITS/ITS_joined_reverse_or_revcomp.fastq
cat ./ITS/ITS_joined_forward_or.fastq ./ITS/ITS_joined_reverse_or_revcomp.fastq >
./ITS/ITS_joined_all_oriented.fastq
```
